# Genome-Wide Characterization of Kiwifruit Invertase Gene Family Reveals Roles of *AcCWINV4* in Sugar Accumulation and Cold Tolerance

**DOI:** 10.3390/ijms262010089

**Published:** 2025-10-16

**Authors:** Aoning Zhang, Xiaomeng Yang, Deshuai Liu, Zhexing Luo, Zhihao Zhang, Junwei Huo, Deguo Han, Yan Zhang, Lihua Zhang

**Affiliations:** The Key Laboratory of Biology and Genetic Improvement of Horticultural Crops (Northeast Region), Ministry of Agriculture and Rural Affairs, National-Local Joint Engineering Research Center for Development and Utilization of Small Fruits in Cold Regions, College of Horticulture & Landscape Architecture, Northeast Agricultural University, Harbin 150030, China; 17339831802@163.com (A.Z.); 13243028138@163.com (X.Y.); lds201220@163.com (D.L.); 17673895464@163.com (Z.L.); zzhh4ever@163.com (Z.Z.); junweihuo@aliyun.com (J.H.); deguohan@neau.edu.cn (D.H.)

**Keywords:** kiwifruit, invertase, sugar accumulation, cold stress

## Abstract

Invertase, a core catalyst in sugar metabolism, irreversibly hydrolyzes sucrose into hexoses, establishing the fundamental biochemical pathway for carbon allocation in plants and playing pivotal roles in plant growth development, fruit quality regulation, and stress response. In the present study, we identified a total of 25 invertase genes from the kiwifruit (*Actinidia chinensis* cv Hongyang) genome and systematically analyzed the physicochemical properties, chromosomal localization, genomic features, and gene evolution patterns of the *AcINV* family. The evaluation of selection pressure indices robustly demonstrated that the *INV* family underwent purification selection during domestication. Furthermore, based on the correlation between gene expression levels during the post-harvest ripening stage of kiwifruit and soluble sugar content, we identified the potential gene *AcCWINV4* as being associated with sugar accumulation. Furthermore, virus-induced gene silencing (VIGS) of *AcCWINV4* confirmed its functional role in fruit sugar accumulation and plant response to cold stress. This study provides critical theoretical support for breeding cold-tolerant and high-quality kiwifruit varieties using molecular biological methods.

## 1. Introduction

Sucrose, as the primary photosynthetic product [1], is involved in various plant physiological processes, including development, plant response, fruit development, and ripening, with dual roles as both a nutrient and a signaling molecule [2,3,4]. During fruit quality development, sweetness, serving as a core sensory attribute, is primarily determined by the dynamic equilibrium and synergistic effects within the sucrose (Suc) metabolic system, encompassing its breakdown products fructose (Fru) and glucose (Glc) [5,6]. Consequently, elucidation of the regulatory mechanisms governing genes involved in fruit sugar metabolism, particularly sucrose metabolism, has emerged as a critical research priority in modern fruit tree breeding and quality enhancement.

Invertase (INV), sucrose phosphate synthase (SPS), and sucrose synthase (SUSY) are crucial enzymes involved in sucrose metabolism and accumulation [7]. INV and SUSY catalyze the hydrolysis of sucrose, while SPS catalyzes the synthesis of sucrose [8]. Among INV isoenzymes, significant functional divergence exists in plants. Specifically, CWINV plays a core role in phloem unloading [3,9], carbon distribution [10,11], sink development [12,13,14], and plant stress response [15] by controlling the source-library sucrose concentration gradient. Similarly, *VINV* has a function closely related to *CWINV*. For example, in tomato, *VINV* is important in regulating sugar metabolism and the postharvest quality of fruits [16], while in cucumber (*Cucumis sativus*), *CsVI2* is involved in sucrose metabolism and regulates drought stress in seedlings [17]. In contrast, unlike CWINV and VINV, the NINV protein is unstable due to a lack of N-terminal signaling peptides and protein glycosylation [18], and thus, there are few related studies. Recent studies have indicated that *NINV11* and *NINV12* are involved in the plant low-temperature response process in apples [19], suggesting their potential role in plant adaptation.

Abiotic stress, especially low-temperature stress, regulates plant development and yield under stress by affecting the plant source–sink relationship [20,21,22,23,24]. When plants are subjected to environmental stress, they increase invertase activity and the expression of their corresponding genes to change the soluble sugar content in cells [8,25]. The *INV* gene expression in oranges (*Poncirus trifoliata*) and apples (*Malus domestica*) will be greatly increased before a low-temperature treatment [19,26]. Sucrose contains multiple hydroxyl groups, which can form non-covalent bonds such as hydrogen bonds with biological macromolecules to stabilize the structural functions of proteins and biofilms [27]. Studies have shown that the accumulation of soluble sugars can increase the stability of cell components and cell membranes at low temperatures [20].

Kiwifruit (*Actinidia chinensis*), a native species of the Actinidiaceae family, is globally cultivated for its distinctive flavor and exceptionally high vitamin C content among fruits [28]. As a climacteric fruit model, it requires postharvest ripening due to its suboptimal texture and sugar–acid ratio at harvest [29]. This process involves more intricate metabolic reprogramming compared to non-climacteric fruits, particularly in soluble sugar dynamics [13]. Deciphering the molecular mechanisms underlying post-ripening sugar accumulation is crucial for quality improvement. Current understanding of the *INV* gene family functions in kiwifruit remains limited, especially regarding their roles in stress responses. The recent release of a high-quality diploid genome assembly now enables genome-wide transcriptomic investigations of sugar-metabolism-related gene families. This genomic resource opens new avenues for characterizing the *INV* family’s dual functions in sugar metabolism and stress adaptation.

In the present study, 25 invertase family genes were identified based on kiwifruit (*Actinidia chinensis*) genome data, and a systematic investigation was performed on the gene structure, phylogenetic clustering, cis-regulatory motifs of its promoter, and molecular evolution characteristics. Furthermore, *AcCWINV4* genes, as key candidate genes for sugar accumulation, were identified via correlation analyses between sugar content and gene expression. Virus-induced gene silencing (VIGS) analysis confirmed the functions of these two candidate genes in fruit sugar accumulation and the low-temperature response of the plant. This work preliminarily elucidates the relationship between *AcCWINV4* genes and hexose accumulation, as well as low-temperature stress responses in kiwifruit, providing crucial theoretical support for breeding cold-resistant and high-quality kiwifruit varieties using molecular biological approaches.

## 2. Results

### 2.1. Identification and Characterization Analyses of the INV Gene Family in Kiwifruit

**Figure 1:**
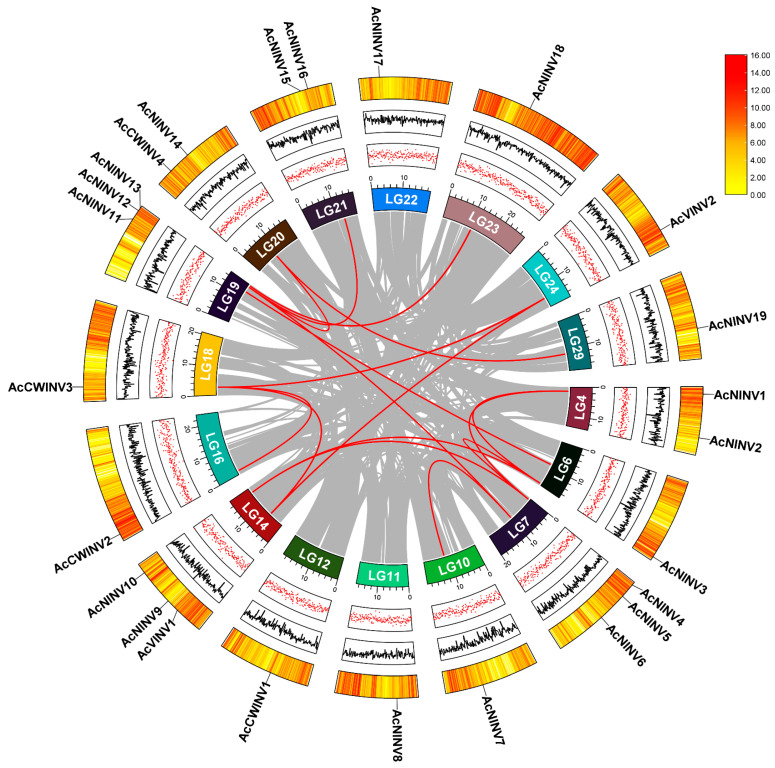
Synteny analysis of *AcINV* genes.

Through homological comparison and identification of conserved structural domains, 19 *NINV* genes, 4 *CWINV* genes, and 2 *VINV* genes were obtained in the kiwifruit genome, namely, *AcNINV1*~*AcNINV19*, *AcCWINV1*~*AcCWINV4*, and *AcVINV1* and *AcVINV2*, respectively (Table S2). Chromosomal localization analysis indicated that 25 *INV* genes were distributed across 16 chromosomes (Figure 1). The physicochemical properties of these *INV* genes showed that the length of their coding sequences (CDSs) varied from 384 to 779 aa, molecular masses (MS) ranged from 42,189.10 to 88,200.76 Da, and the theoretical isoelectric point (pI) varied from 4.75 to 9.1 (Table S2). Furthermore, predictions of protein subcellular localization showed that the *AcINV* family is predominantly distributed in chloroplasts and cytoplasm (Table S2).

A multiple collinearity analysis of the *INV* gene family and a circle plot diagram are shown in Figure 1. This study observed 25 genes distributed across multiple loci on 16 chromosomes, with three cases of tandem repeats observed on chromosomes LG7, 14, and 19, indicating that these regions are hotspots for gene duplication events. Twenty pairs of multiple collinearity genes were analyzed in the *INV* gene. To fully elucidate the evolutionary relationships within the kiwifruit *INV* gene family, INV proteins from *Arabidopsis*, grapes, and kiwifruit were selected to construct a phylogenetic tree. Cluster analysis results (Figure S1) showed that the AINV and NINV proteins from the three species were located on different branches, suggesting that they exhibit significant differences in catalytic properties and physiological functions, likely resulting from functional specialization under long-term natural selection.

### 2.2. Structure and Selection Pressure Analyses of AcINVs

**Figure 2:**
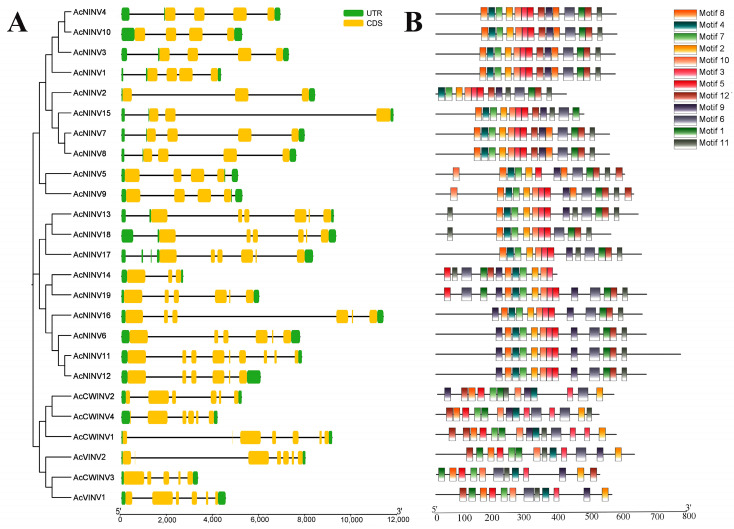
Analysis of the structure and conserved motifs of the kiwifruit *INV* family genes. (**A**) Analysis of the structure of the *INV* family of genes in kiwifruit. (**B**) Conserved motif analysis of kiwifruit *INV* family genes.

The Gene Structure Display Server (http://gsds.cbi.pku.edu.cn/) (accessed on 1 September 2024) was used to analyze the structures of *AcINV* genes according to their CDS and contrasted genomic sequences. The results indicate that *AcVINV* and *AcCWINV* possess 5 to 7 exons, while the *AcNINV* gene ranged from 3 to 9 (Figure 2A). To further understand the *AcINV* structure, protein conserved motifs were analyzed with the MEME server (http://meme-suite.org/). We found that *AcINV* family members all have the 12 putative conserved motifs, and the lengths of the identified conserved motifs varied from 15 to 50 (Figure 2B, Table S3). The core function of motif 1 is to constitute the active center of the enzyme responsible for sucrose hydrolysis and to maintain the structural stability of the enzyme, directly determining *INV*’s core ability to break down sucrose. Motif 7 serves as the pivotal structural unit within the *INV* gene family for executing sucrose hydrolysis. Its core function lies in constructing the substrate-binding pocket and maintaining the spatial conformation essential for enzyme catalysis, thereby acting as the functional scaffold that ensures the efficient exercise of *INV* enzyme activity. Motif 7 is widely conserved within the *INV* gene subfamily.

Two types of gene replication occur during plant evolution, recent replication (duplication events within the same species) and old replication (duplication events between different species) [30]. According to the evolutionary relationship shown in Supplementary Table S3, three old duplication events and six recent duplication events were noted in the *INV* gene. It is worth noting that “nested old repetitive events” were observed in the *NINV* subfamily, indicating that this subgroup experienced at least one duplication event before species radiation.

Syntenic collinearity identified twenty duplicated gene pairs. Interestingly, the *AcNINV6*/*AcNINV7* pair was derived from whole-genome or segmental duplication, while the *AcNINV6*/*AcNINV12* pairs were derived from random duplication events (Figure 1), indicating that whole-genome or segmental duplication and random duplication might be the major driving forces for *AcINVs’* expansion. Furthermore, we evaluated the evolutionary selection pressure on homologous gene pairs by calculating the Ka/Ks (nonsynonymous–synonymous substitution ratio) (Supplementary Table S4). The results indicate that the Ka/Ks ratios for all gene pairs were significantly less than 1, suggesting that the *INV* gene family underwent intense purifying selection during evolution. Its function tends toward preserving ancestral traits or becoming more robust, rather than undergoing significant innovative differentiation.

### 2.3. Expression Patterns of AcINV Family Gene in Source and Sink Tissues

*INV* is capable of irreversibly catalyzing the hydrolysis of sucrose to regulate the unloading and distribution of soluble sugar in sink tissues [8]. To analyze the expression patterns of *AcINV* family genes in kiwifruit source–sink tissues, RNA-seq data of the root, stem, new leaves, mature leaves, and fruits were analyzed. Several *AcINV* genes showed significantly different expression levels in the above tissues (Figure S2). Among them, *NINV1/3/15/17* and *AcCWINV2* exhibited high abundance in leaf tissues. Collectively, the vast majority of *INV* genes showed preferential expression in fruit tissues, suggesting that *AcINV* family genes may play key roles in fruit development and quality formation. 

**Figure 3:**
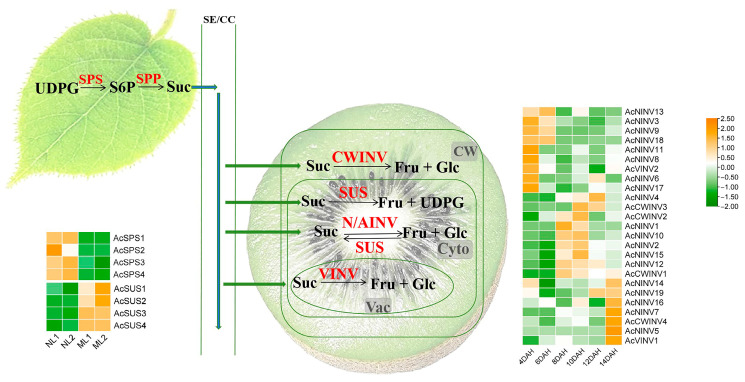
Sketch of kiwifruit sugar metabolism pathways and heat map of sugar metabolism gene expression profiles at fruit developmental stages.

To better understand the expression patterns of *AcINVs* in fruits, we analyzed the transcriptional abundance differences of *AcINV* family genes in kiwifruit at six different time points (4DAH, 6DAH, 8DAH, 10DAH, 12DAH, and 14DAH). The results indicated that the expression changes in all *INV* family genes during fruit maturation could be categorized into three types: initially increasing and then decreasing, gradually increasing, and gradually decreasing. We focused on *AcNINV7*, *AcCWINV4*, and *AcVINV1*, as these three genes exhibited gradually increasing expression levels during fruit maturation and had high expression abundance in mature fruits (Figure 3), suggesting they may play a key role in kiwifruit maturation and sugar accumulation.

### 2.4. Correlation Analysis Between INV Expression and Sugar Content

To analyze the relationship between *AcINV* gene expression levels and sugar contents during kiwifruit storage, we first measured the contents of main soluble sugars, including sucrose (Suc), fructose (Fru), and glucose (Glc), in kiwifruit at various storage stages. Among these soluble sugars, the contents of glucose and fructose generally show an upward trend. As the fruit ripens, the sucrose content shows a trend of first increasing and then decreasing, reaching its peak at 10DAH (Figure S3). The total sugar content gradually increases as the fruit ripens, reaching its maximum at 10 DAH, after which it declines.

**Figure 4:**
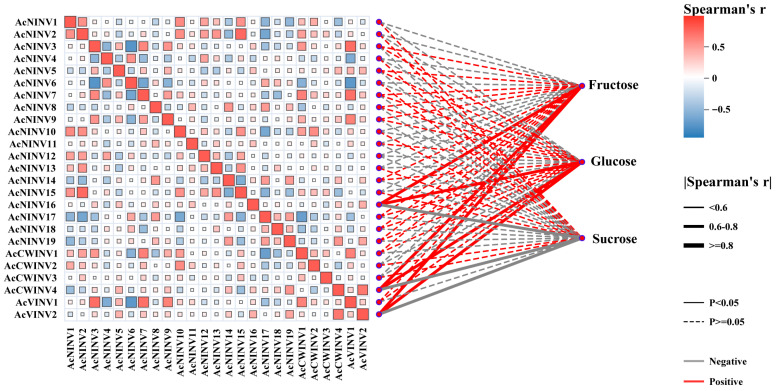
Correlation analysis between the expression level of the *INV* gene in kiwifruit and soluble sugar content. Solid line represents *p* < 0.05, dashed line represents *p* ≥ 0.05, gray line represents negative correlation, and red line represents positive correlation.

To analyze the relationship between *AcINV* gene expression and sugar accumulation patterns during kiwifruit fruit ripening, Pearson correlation coefficient (PCC) analysis was carried out and is summarized in Figure 4. Among the genes *AcNINV7*, *AcCWINV4*, and *AcVINV1*, which are highly expressed in mature fruit as mentioned above, the expression pattern of *AcCWINV4* shows a significant negative correlation with sucrose, suggesting *AcCWINV4* may play a crucial role in regulating sugar content during kiwifruit ripening.

### 2.5. Functional Analysis of AcCWINV4 Regarding Sugar Contents

**Figure 5:**
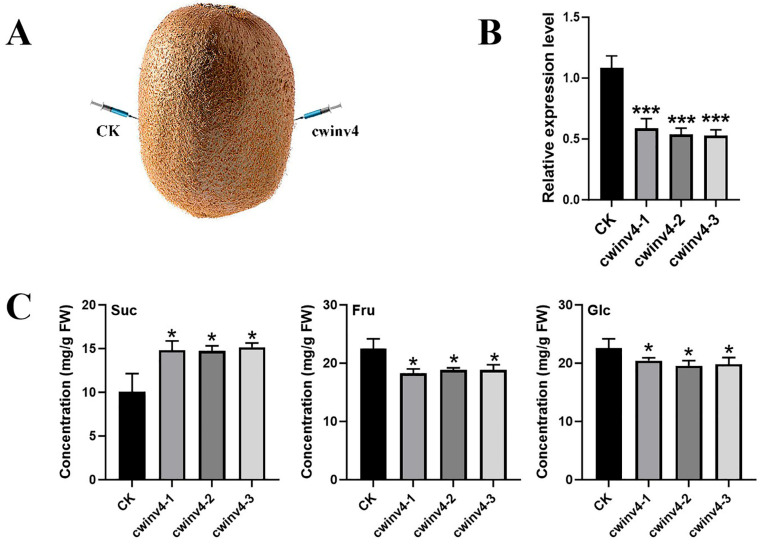
*AcCWINV4* gene silencing to analyze the soluble sugar content of kiwifruit fruit. (**A**) *AcCWINV4*-gene-silenced fruit phenotypes. (**B**) Levels of *AcCWINV4* in fruits where the *AcCWINV4* genes were silenced. (**C**) *AcCWINV4* gene silencing of glucose, fructose, and sucrose content in fruits. * Represents *p* < 0.05, and *** represents *p* < 0.001.

To investigate the role of *AcCWINV4* in sugar accumulation during fruit ripening, the expression of *AcCWINV4* was silenced in kiwifruit fruit using the VIGS technique (Figure 5A). Compared with control fruits, the expression of *AcCWINV4* was significantly reduced in *Accwinv4*-silenced fruits, while fructose and glucose content were significantly reduced, and sucrose content was significantly increased (Figure 5B,C). These results validate our hypothesis that *AcCWINV4* plays an inhibitory role in sucrose accumulation.

### 2.6. The Expression of AcCWINV4 Is Induced by Low Temperature

To elucidate whether the *AcINV* family genes are induced by environmental stress, we systematically characterized the cis-acting elements within their promoter regions, with a focus on *AcCWINV4*. The results showed that the functions of cis-elements are mainly related to the response to light, hormones, and abiotic stress (Figure S4). The diversity of cis-acting elements in *AcINV* promoters suggested potential functional roles in multiple biological processes, including plant development, hormone response, and abiotic stress. It is worth noting that the promoter regions of *AcCWINV4* contain three stress response elements and four low-temperature response components, indicating its potential functions in response to external environmental stress, especially low-temperature stress.

**Figure 6:**
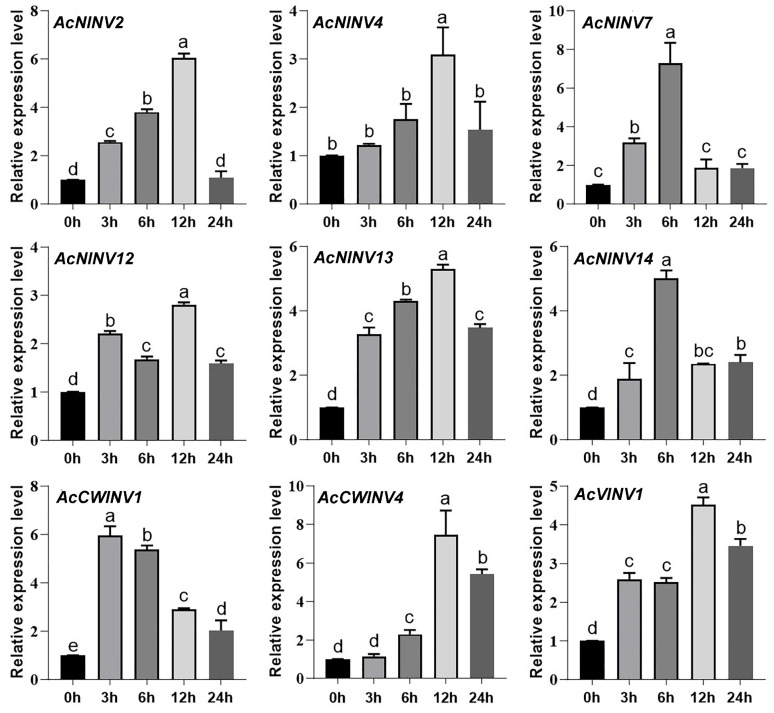
The expression patterns of *AcINV* genes under cold treatment. a, b, c, d, etc., denote the significance of differences between groups. Identical letters indicate no significant difference between groups (*p* > 0.05), while different letters indicate significant differences between groups (*p* < 0.05).

Studies have highlighted that the accumulation of soluble sugar in plants could enhance the ability of plants to resist low-temperature stress, and the relationship between the expression of essential genes related to sucrose accumulation, such as *SWEET*, *NINV*, *AINV*, and plant cold resistance, has been studied [19,31], while the transcription expression of *AcCWINV4* genes under cold stress has rarely been reported. Here, qRT-PCR analysis was carried out to evaluate the expression profiles of *AcINV* family members in kiwifruit leaves under cold conditions. The expression levels of the tested *INV* genes were all induced by low temperature, but there were differences in the time of rapid response to low temperature. The expression of *AcCWINV4* was rapidly upregulated after 6 h of low-temperature treatment and reached its peak at 12 h (Figure 6), indicating its potential function in plant low-temperature response.

### 2.7. Functional Analysis of AcCWINV4 Regarding Cold Tolerance

**Figure 7:**
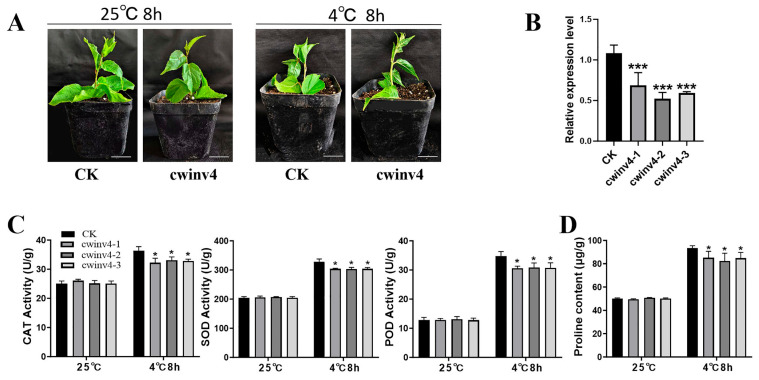
Cold resistance analysis of leaves of kiwifruit with *AcCWINV4* gene silence. (**A**) Phenotypes of CK and *cwinv4* at 25 °C and 4 °C for 8 h, respectively. (**B**) Expression level of *AcCWINV4* after silencing of *AcCWINV4* gene. (**C**) The activities of SOD, POD, and CAT in CK and *cwinv4* were analyzed after 8 h at 25 °C and 4 °C, respectively. (**D**) The proline content of CK and *cwinv4* was determined after being left at 25 °C and 4 °C for 8 h, respectively. * Represents *p* < 0.05, and *** represents *p* < 0.001.

To investigate the function of *AcCWINV4* under cold stress, a virus-induced silent vector was constructed by referring to the method of Huimin Zhou [32] and transferred into the leaves of kiwi seedlings. Three transgenic lines (*cwinv4-1*, *2*, *3*) were generated and used for cold treatment. Compared with the control plants (CK), *Accwinv4*-silenced plants showed lower expression levels of *AcCWINV4* (Figure 7B). After 8 h of low-temperature treatment at 4 °C, the leaves of *Accwinv4-*silenced plants wilted, while the leaves of CK plants grew well (Figure 7A).

In plants, peroxidase (POD) and catalase (CAT) play an important role in removing reactive oxygen species (ROS) [19,33]. Furthermore, the activities of antioxidant enzymes in the leaves of transgenic and control plants under the low-temperature treatment were analyzed. The results showed that *Accwinv4*-silenced leaves generate reduced antioxidant enzyme activity in CAT, POD, and SOD compared with CK under cold stress (Figure 7C). In addition, proline is a core osmotic regulatory substance for plants to respond to abiotic stress [34]. After the low-temperature treatment, the transgenic plants had reduced proline content compared with the control (Figure 7D).

## 3. Discussion

*INVs* play crucial regulatory roles in plant physiological processes by catalyzing the irreversible hydrolysis of sucrose into glucose and fructose, thereby influencing growth, development, and fruit sugar accumulation in higher plants [8,9,15]. As key modulators of sugar content, the *INV* gene family has been systematically characterized in the model plants *Arabidopsis* [23] and tomatoes [35]; main crops such as rice [36]; strawberry [37], passionfruit [14], and apples [19]; and pepper [38] and cassava vegetables [39]. Despite extensive knowledge of *INV*-mediated sucrose hydrolysis, its functional roles in abiotic stress adaptation in kiwifruit remain unclear. In this study, we identified and demonstrated the roles of *AcCWINV4* in sugar accumulation and cold stress responses in kiwifruit, laying a foundation for improving fruit quality and stress resistance.

Conservative motif analysis serves as a crucial tool for elucidating the evolutionary origins and phylogenetic relationships of genes, revealing functional constraints and evolutionary trajectories of gene families [3,40]. In this study, conserved motifs were identified across all three *INV* families (Figure 2B), indicating the evolutionary conservation of *AcINV* genes. Phylogenetic analysis based on *INV* sequences from kiwifruit, grapes, and *Arabidopsis thaliana* demonstrated that this gene family can be clearly classified into four subfamilies (*CWINV*, *VINV*, *NINV*-α, and *NINV*-β) (Figure S1), which is consistent with apple and tomato species [19,35]. Structural variations within subfamilies may determine their functional diversity. Synteny analysis revealed that both whole-genome or segmental duplication events and random duplications collectively drive the genomic evolution and family expansion of *AcINV*, aligning with previous studies on strawberry and apple [19,37]. The ratio of non-synonymous (Ka) to synonymous (Ks) substitution rates generally serves as an indicator of selective pressure on genes or genomic regions [41]. Further Ka/Ks analysis (all ratios < 1, Table S4) indicated that the *INV* family underwent purifying selection after duplication events, potentially promoting functional divergence among gene copies. The core functionalities encoded by genes responsible for sucrose degradation are rigorously preserved, ensuring a stable supply of fundamental carbon sources within the plant’s sugar metabolic pathways. This prevents critical metabolic steps from failing due to genetic variation. Even when minor mutations occur, they are predominantly neutral mutations that do not affect enzyme activity, allowing the gene as a whole to maintain its original function.

During fruit development, the dynamic expression of sugar metabolism-related genes governs changes in soluble sugar content. In passionfruit, PeCWINV5 expression progressively increases, paralleling fructose and glucose accumulation throughout fruit development and ripening, and functional characterization further reveals its positive regulatory role in hexose accumulation [14]. Similar phenomena have also been observed in strawberries (Fragaria ananassa) and grapes (Vitis vinifera) [37,42]. Pearson correlation coefficients (PCCs) indicate a negative correlation between AcCWINV4 expression levels and sucrose content during the post-ripening process of “Hongyang” kiwifruit (Figure 4), suggesting that AcCWINV4 likely plays a significant role in sucrose accumulation.

Invertases are classified into acid invertases (CWINV, VINV) and neutral invertases (NINV) based on their optimal pH [43]. Acid invertases play pivotal roles in carbon partitioning [8,10] and sink organ development [14], regulating the source–sink sucrose concentration gradient [44]. Therein, the role of *CWINV* in fruit sugar accumulation has been well documented in apple, passion, Citrus, and other fruits, where most *CWINVs* catalyze the conversion of sucrose to hexoses [13]. Consistent with the above findings, silencing *AcCWINV4* in kiwifruit increased sucrose content while reducing fructose and glucose accumulation (Figure 5). It is hypothesized that *AcCWINV4* plays a crucial role in the extracellular hydrolysis of sucrose and the supply of monosaccharides in kiwifruit.

The accumulation of soluble sugar is intrinsically linked to plant cold resistance, which is a significant agronomical trait in cultivated kiwifruit [20,27]. Differences in specific promoter regions of a gene critically influence gene function [3]. Promoter analysis of AcCWINV4 showed multiple low-temperature responsive cis-elements, and its expression was significantly induced under cold stress (Figures S4 and 6), indicating its potential role in cold resistance responses. Furthermore, silencing AcCWINV4 substantially reduced plant cold tolerance, and transgenic lines exhibited markedly decreased antioxidant enzyme activities and proline accumulation (Figure 7). These findings demonstrate that *Accwinv4*-silenced plants enhance ROS accumulation by diminishing the antioxidant capacity of kiwifruit plants, thereby reducing kiwifruit cold tolerance. Similar results have been observed in passionfruit and Citrus [14,15]. The above results indicate that AcCWINV4 is involved in the process of kiwifruit plants responding to low-temperature stress. Fructose and glucose, as small-molecule osmotic regulators, enhance cold tolerance by elevating cellular osmotic pressure and stabilizing cell membrane structure. *AcCWINV*4 silencing reduces their production, depriving plants of this “cold-protective substance” and directly manifesting as diminished cold resistance. While sucrose serves as an energy storage compound, it cannot directly and efficiently perform osmotic regulation; its accumulation cannot compensate for the cold-tolerance deficit caused by monosaccharide deficiency.

## 4. Materials and Methods

### 4.1. Plant Material and Processing Methods

“Hongyang” kiwifruit (*Actinidia chinensis*) plant was grown on a farm in Yangling City, Shaanxi Province, China (34°20′ N, 108°24′ E). In this study, we collected roots, stems, young leaves, mature leaves, and fruits from “Hongyang” kiwifruit for tissue-specific expression analysis. After harvesting, fruits were stored at room temperature for 4, 6, 8, 10, 12, and 14 days (DAH). Samples were collected and frozen in liquid nitrogen for subsequent determination of sugar content and gene expression levels. All experiments were conducted with three biological replicates [45].

The kiwifruit plants were cultured in a medium containing MS medium, 30 g/L sucrose, 0.1 mL IAA (1 mg/mL), and 0.2 mL ZT (1 mg/mL), with the pH adjusted to 5.8. The plants were cultured at 24 °C under a photoperiod of 16 h of light and 8 h of darkness, with a light intensity of 2000 Lux. After 50 days, the seedlings were transplanted into solid medium (peat moss: perlite = 1:1) for subsequent experiments. Select healthy seedlings exhibited uniform growth for low-temperature treatment at 4 °C. Samples were collected at 0, 3, 6, 12, and 24 h post-treatment, with three replicates per experiment. Samples were rapidly frozen in liquid nitrogen and stored at −80 °C [46].

### 4.2. Identification of Kiwifruit INV Gene Families

Screening of kiwifruit *INV* candidate genes: Kiwifruit *INV* candidate genes were obtained by homologous comparison with the amino acid conserved protein sequences of the *Arabidopsis thaliana INV* gene in the kiwifruit (*Actinidia chinensis* cv Hongyang 1.0) database (http://kiwifruitgenome.org/home) (accessed on 22 August 2024). Target genes were screened via BLAST (http://kiwifruitgenome.org/home) (accessed on 22 August 2024) alignment using an E-value threshold of ≤1 × 10^−5^. DNAMan 6.0.40 was used to screen the sequences of the above candidate genes, and the duplicate sequences were eliminated. Then, the conserved sequences of *INV* genes were screened by NCBI (https://www.ncbi.nlm.nih.gov/Structure/bwrpsb/bwrpsb.cgi) (accessed on 22 August 2024), and the candidate genes that did not contain specific structural domains were deleted. The remaining *INV* gene families underwent physicochemical property analysis using the Expasy online tool (https://web.expasy.org/protparam/) (accessed on 23 August 2024). Protein secondary structure was analyzed using PRABI (https: //www.predictprotein.org/) (accessed on 25 August 2024), and subcellular localization was predicted using the online software WOLF PSORT (https: //wolfpsort.hgc.jp/) (accessed on 26 August 2024) [47].

### 4.3. Bioinformatic Analysis of the INV Gene Family in Kiwifruit

Based on database information from the kiwifruit genome (http://kiwifruitgenome.org/home) (accessed on 27 August 2024), the location of *AcINVs* in the kiwifruit chromosome was determined, and intraspecific synteny analysis was performed using the TBtools (https://bio.tools/tbtools) (accessed on 28 August 2024) software. Phylogenetic trees of INV protein sequences from various species, including kiwifruit, *Arabidopsis*, and grapes, were constructed using the MEGA 7.0 software. The structure of the kiwifruit *INV* gene was analyzed using the online software GSDS (http://gsds.cbi.pku.edu.cn/) (accessed on 1 September 2024), and the conserved motifs in kiwifruit INV proteins were identified using the online software MultipleEmforMotifElicitation (MEME) Version 5.0.5 (http://meme-suite.org/tools/meme) (accessed on 2 September 2024) to identify conserved motifs (set to 12 motifs) in the kiwifruit INV protein. The TBtools software was used for motif visualization. The online website PlantCARE (http://bioinformatics.psb.ugent.be/webtools/plantcare/html/) (accessed on 3 September 2024)was used to analyze the 2000-base pair upstream promoter sequence of the kiwifruit *INV* gene family. The MCS-canX (accessed on 4 September 2024)software was used to identify collinearity between *Arabidopsis* and kiwifruit *INV* genes, with a threshold of E ≤ 1 × 10^−5^, to find large-scale duplicate gene pairs and tandem repeat gene pairs. The Ka and Ks values of collinear gene pairs were then calculated using the YN algorithm in the KaKs_Calculator (2.0) toolkit [19].

### 4.4. Gene Silencing of AcCWINV4

VIGS-mediated gene silencing was used to silence the expression of *AcCWINV4* in kiwifruit. Gene-specific fragments (*AcCWINV4*: 261 bp) of *AcCWINV4* (Table S1) were first selected and amplified by RT-PCR and subsequently constructed into the pTRV2 vector and transformed into Agrobacterium GV3101. The bacteriophage containing pTRV2-*AcCWINV4* was incubated separately to an OD_600_ of about 1.0 and resuspended with pTRV1 at a 1:1 ratio to an OD_600_ of 0.4–0.6. pTRV2 empty vector was used as a control, and the mixture was injected in four directions perpendicular to the longitudinal diameter of the middle of the kiwifruit using a 1 mL syringe. We selected four positions at the equator of each fruit and slowly injected 400 μL of the mixture in the direction of the center to a depth of 0.2 cm, with half of the same fruit injected with the target gene and the other half with the empty vector. It was placed in a constant-temperature incubator at 25 °C. On the third day of dark treatment, fruit samples were collected, rapidly frozen in liquid nitrogen, and subsequently analyzed for target gene expression levels and soluble sugar content. The experiment was conducted in triplicate [48].

The kiwifruit seedlings were gently removed from the soil mixture (without causing root damage). All kiwifruit leaves were immersed in the above resuspension solution (roots were wrapped with tinfoil to protect them) and placed in a plastic vacuum drier, connected to a vacuum pump, and pumped under vacuum for about 8 min until the area of infested leaves reached 80% of the foliage surface. Then, the vacuum pump was quickly switched off. The treated kiwifruit seedlings were transferred back to the soil mixture, and the culture box was wrapped with cling film to keep it moist. Three replicates were set up for each group of seedlings, which were incubated in a constant-temperature incubator at 25 °C for 3 days in the dark, and the transcript levels of the target genes in the leaves of the transformed plants were examined by qRT-PCR [32].

### 4.5. Determination of Sugar Content

The soluble sugar content of kiwifruit fruit was determined using a Shimadzu gas chromatograph GC-MS-2010SE (Shimadzu Corporation, Tokyo, Japan). Frozen samples were weighed to the nearest 0.1 g, dispensed into pre-cooled 1.5 mL capped centrifuge tubes, and the weighed mass was recorded accurately. In total, 1.4 mL of pre-cooled 75% methanol was rapidly added to each sample, along with 0.1 mL of Ribitol (400 ppm), and the samples were centrifuged for 10 min in a metal bath (70 °C, 950 rpm for 30 min) at a rotational speed of 12,000 rpm at room temperature. After centrifugation for 10 min, the supernatant was pipetted into a pre-prepared and dried 10 mL glass centrifuge tube, and 0.75 mL of pre-cooled chloroform and 1.4 mL of H_2_O were added, vortexed and shaken, and centrifuged at 2200× *g* for 15 min; then, 1 mL of the supernatant was aspirated into a 1.5 mL silica centrifuge tube and stored at −80 °C, to be used later. After the sample was thawed and vortexed, 5 μL of the sample was aspirated into another siliconized centrifuge tube and dried under vacuum for 1 h, 40 μL of methoxylate was added and placed in a metal bath (37 °C, 950 rpm, 2 h), 60 μL of MSTFA was added and placed in a metal bath (37 °C, 300 rpm, 30 min); the sample was pipetted into a small brown vial containing a liner tube, stored at room temperature, and protected from light, and the GC-MS on-board measurement was performed [6].

### 4.6. RNA Extraction and qRT-PCR Analysis

The five different tissues harvested, including roots, stems, new leaves, mature leaves, and fruits, as well as treated kiwifruit fruits and leaves, were subjected to RNA extraction using the Plant RNA Extraction Kit (Vazyme, Nanjing, China) and cDNA synthesis using a Reverse Transcription Kit (TRAN, Beijing, China). qRT-PCR reactions were performed using Taq DNA polymerase (TaKaRa, Dalian, China), with primers listed in Table S1 and repeated three times. The relative expression of the candidate genes was calculated by using the 2^−∆∆Ct^ method [49,50].

### 4.7. Measurement of Cold Resistance

SOD (superoxide dismutase), POD (peroxidase), CAT (catalase), and Pro (proline) contents were determined in transgenic kiwifruit leaves at 25 °C and 4 °C for 8 h [51,52], as described in Lihua Zhang [34].

### 4.8. Statistical Analysis

Statistical analysis was performed using one-way ANOVA (IBM SPSS Statistics 21) with significance set at *p* < 0.05. Data are presented as mean ± SD from three biological replicates [53].

## 5. Conclusions

This study systematically characterized the *INV* gene family in kiwifruit and elucidated its critical functions in fruit sugar accumulation and plant cold resistance. A total of 25 *AcINV* genes were identified, and comprehensively we described their gene and promoter structures, physicochemical properties, chromosomal locations, and evolutionary patterns. Analysis of sugar content and *AcINV* gene expression across different postharvest ripening stages of kiwifruit indicated the potentially important function of *AcCWINV4* on sucrose accumulation. Furthermore, *AcCWINV4* expression is induced by low temperatures, and the low-temperature tolerance of *Accwinv4*-silenced kiwi plants was significantly reduced. These findings establish a foundation for further elucidating the regulatory functions of *AcINV* family genes in fruit development and low-temperature stress adaptation, providing a theoretical basis for cultivar improvement and molecular breeding.

## Data Availability

The original contributions presented in this study are included in the article/Supplementary Material. Further inquiries can be directed to the corresponding authors.

## Supplementary Materials

The following supporting information can be downloaded at https://www.mdpi.com/article/10.3390/ijms262010089/s1.
