# Supplementary material for: Genome-Wide Characterization of Kiwifruit Invertase Gene Family Reveals Roles of AcCWINV4 in Sugar Accumulation and Cold Tolerance
Source: Int J Mol Sci. 2025 Oct 16;26(20):10089. doi: 10.3390/ijms262010089 (PMC12562396; doi:10.3390/ijms262010089)
Supplement: Supplementary file 1 [file ijms-26-10089-s001.zip › ijms-3866626-supplementary.pdf]

Table S1. Primer sequences used for RT-qPCR.

Gene	Gene ID	New Gene ID	Forward primer(5'-3')	Reverse primer(3'-5')
pTRV-*AcCWINV4*	*Acc22611*	*Achrdv1x04g045730*	CGATCCTTCCAGGCGAGACA	TTGCAGTTCCGTGACCGTTA
AcNINV1-qRT	*Acc04550*	*Achrdv1x04g040100*	CGAGACTCTACGCGCGGATT	ACGTGTCACCCGTGGACTTC
AcNINV2-qRT	*Acc05177*	*Achrdv1x05g054590*	TGGTTCGCGTTGGGTACTTG	ACTACCTCTTCCCAGCGTGC
AcNINV3-qRT	*Acc07110*	*Achrdv1x06g061230*	TGCATCACGACGGGACGAC	GTTTACGAGCCTGCTTCCCT
AcNINV4-qRT	*Acc07278*	*Achrdv1x06g066420*	TCACTTGGGTTGTCTCCGCA	CGTCGACCTGGTGACAGTGT
AcNINV5-qRT	*Acc07563*	*Achrdv1x07g067360*	ACTGTCGCCGGACACGCACC	TTCTCGTCTGCGATTGTCGG
AcNINV6-qRT	*Acc08050*	*Achrdv1x07g070050*	ACTGTGGTGGTGCCACATGT	TTGGGCTCCCACACTACGTT
AcNINV7-qRT	*Acc11463*	*Achrdv1x07g073690*	GATATGTCGGCACGCACGCA	GCTTGTCCTCTTCCAGAGCG
AcNINV8-qRT	*Acc12236*	*Achrdv1x10g107220*	GGCCGGACTATTACGACGGT	CGACGGATCCTCCACCATCA
AcNINV9-qRT	*Acc15916*	*Achrdv1x10g107720*	GCACCATTCATCGCCGGACG	AGTACGAGCTCCGTAGCCGA
AcNINV10-qRT	*Acc16258*	*Achrdv1x11g115800*	GCCTCCACGGCCGTTGACTA	GTCGACCCGGTGACAGAGG
AcNINV11-qRT	*Acc21322*	*Achrdv1x12g134320*	TTCGGACCTCTGGACGGTTG	ACGATGAGCATACCCTGCGA
AcNINV12-qRT	*Acc21472*	*Achrdv1x12g129500*	ATTCACCGCCGACACTCTGC	ACCCCACACTCCGAGGCCAC
AcNINV13-qRT	*Acc21526*	*Achrdv1x13g143110*	ACCAGGCTGATAGCGTGGAC	ACTGTTGAGCCACGTCACCT
AcNINV14-qRT	*Acc22907*	*Achrdv1x13g135020*	ACCACCTCCATTTTCGGGTTT	CCACCTCGACTCGGACTCACC
AcNINV15-qRT	*Acc24121*	*Achrdv1x14g154650*	CGCTGGGAGGAGTTAGTTGG	GCTTGACGGACGACCCTGGC
AcNINV16-qRT	*Acc24210*	*Achrdv1x14g153350*	TGGCCACCCCTTTTATGGCA	ACTATCCACCGGACGCCTCT
AcNINV17-qRT	*Acc24629*	*Achrdv1x14g149380*	ATCCATGGTCACCCTCTGGA	AGTGCTTGGATCAGGTCAGC
AcNINV18-qRT	*Acc26235*	*Achrdv1x16g181400*	TTGCCGACACTGCCGTCACG	TCGTGCCTGTTTTCCGATGA
AcNINV19-qRT	*Acc33185*	*Achrdv1x16g170550*	TGGGACACAGACTTTGATTCACG	CAGGATCACTAGCGGCACCC
AcCWINV1-qRT	*Acc13406*	*Achrdv1x18g202340*	GTGGGTTTGGTGTTGTACGG	CCAGTGCTTTTTGGGCTGAC
AcCWINV2-qRT	*Acc17686*	*Achrdv1x19g208180*	TCAGGCCACTACCCCAGACA	ATGGGCCCACACTATGTTCC
AcCWINV3-qRT	*Acc19909*	*Achrdv1x19g209650*	CACGTGGCGGTTCACGATTG	CCACACACTCCCACATACCC
AcCWINV4-qRT	*Acc22611*	*Achrdv1x19g210190*	TGCAGTGATCAGAGCAGGTC	CAGTGATGCATGTGACCCCT
AcVINV1-qRT	*Acc15785*	*Achrdv1x20g212800*	GTAGCGTTGTTGTTGGATTGGT	TGGCATTCCTACACGCACCC
AcVINV2-qRT	*Acc27954*	*Achrdv1x20g215780*	GCTCTCGTCTTTGGTCGCTT	CCGGAGTATTCCCTGACGGC
AcSPS1-qRT	*Acc06472*	*Achrdv1x21g224410*	GTGGCAGCATGTAGAATGCG	TGCACGTCCTTGAGTGAGTC
AcSPS2-qRT	*Acc11414*	*Achrdv1x21g223630*	CGGGTACTTGGAGGCGATTT	TGTCCTGTGGAGGTCAGACT
AcSPS3-qRT	*Acc14495*	*Achrdv1x22g240090*	GCAAGGGCTTTGGGTTCAAT	TGGAATACGAATAATATAAGCGCCA
AcSPS4-qRT	*Acc18783*	*Achrdv1x23g257390*	ACCACAAGCAGTCTGACGTT	TGATCCACATCCAACCAGCC
AcSUS1-qRT	*Acc05333*	*Achrdv1x24g274380*	CCGATTGCCAACACAAAGCA	GTCCGTAGCGTCCTGTTGAT
AcSUS2-qRT	*Acc12922*	*Achrdv1x28g317220*	ATTGCTCACGCCTTGGAGAA	GAGTCCGGGCATAGTGAAGG
AcSUS3-qRT	*Acc15261*	*Achrdv1x29g326890*	CGAGAGACGTGAAACTTTGCG	CCCATCAATTGCTAGAGGGACA
AcSUS4-qRT	*Acc32312*	*Achrdv1x04g045730*	TCTCAAGCAGGTCTCAAGCG	TGAAGCATGTTTGAAACCACGG

Table S2. *AcINV* gene familiy information in kiwifruit.

Gene	Gene ID	Ac	MW（Da）	PI	Alpha helix (%)	Beta turn (%)	Random coil (%)	Subcellular localization
AcNINV1	*Acc04550*	570	65295.64	6.24	44.04%	2.98%	52.98%	chloroplast
AcNINV2	*Acc05177*	414	47354.87	7.07	49.28%	4.59%	46.14%	cytoplasm
AcNINV3	*Acc07110*	570	64830.46	6.06	42.28%	6.14%	51.58%	cytoplasm
AcNINV4	*Acc07278*	574	65692.32	6.02	41.64%	5.57%	52.79%	chloroplast
AcNINV5	*Acc07563*	601	67786.32	6.43	42.43%	4.49%	53.08%	cytoplasm
AcNINV6	*Acc08050*	669	76551.68	6.67	47.38%	4.33%	48.28%	mitochondria
AcNINV7	*Acc11463*	552	63017.53	6.31	47.28%	5.07%	47.64%	chloroplast
AcNINV8	*Acc12236*	552	62907.35	6.07	45.65%	4.53%	49.82%	chloroplast
AcNINV9	*Acc15916*	630	70724.9	5.88	45.24%	5.08%	49.68%	nucleus
AcNINV10	*Acc16258*	576	65689.38	6.08	44.97%	5.21%	49.83%	cytoplasm
AcNINV11	*Acc21322*	779	88200.76	6.18	51.22%	7.19%	41.59%	mitochondria
AcNINV12	*Acc21472*	669	76421.31	6.35	42.75%	6.58%	50.67%	chloroplast
AcNINV13	*Acc21526*	644	72510.88	5.88	45.34%	6.83%	47.83%	chloroplast
AcNINV14	*Acc22907*	384	42189.10	6.13	26.82%	10.94%	62.24%	chloroplast
AcNINV15	*Acc24121*	470	53530.34	5.43	44.47%	7.66%	47.87%	chloroplast
AcNINV16	*Acc24210*	657	74900.43	5.99	45.81%	6.09%	48.10%	chloroplast
AcNINV17	*Acc24629*	654	73445.1	5.87	44.04%	7.19%	48.78%	chloroplast
AcNINV18	*Acc26235*	556	61822.65	6.37	45.68%	5.22%	49.10%	chloroplast
AcNINV19	*Acc33185*	671	75595.41	6.21	43.07%	4.32%	52.61%	chloroplast
AcCWINV1	*Acc13406*	574	64717.52	8.	12.02%	26.48%	61.50%	chloroplast
AcCWINV2	*Acc17686*	566	63808.96	9.1	11.84%	29.15%	59.01%	vacuole
AcCWINV3	*Acc19909*	521	58437.46	4.75	8.06%	27.83%	64.11%	chloroplast
AcCWINV4	*Acc22611*	519	58846.45	5.88	7.51%	28.13%	64.35%	nucleus
AcVINV1	*Acc15785*	560	63158.34	5.02	12.50%	28.21%	59.29%	chloroplast
AcVINV2	*Acc27954*	632	70308.62	4.95	11.23%	24.53%	64.24%	nucleus

Ac: Amino Acids; MW: Molecular weight of the amino acid sequence; pI: Theoretical isoelectric point.

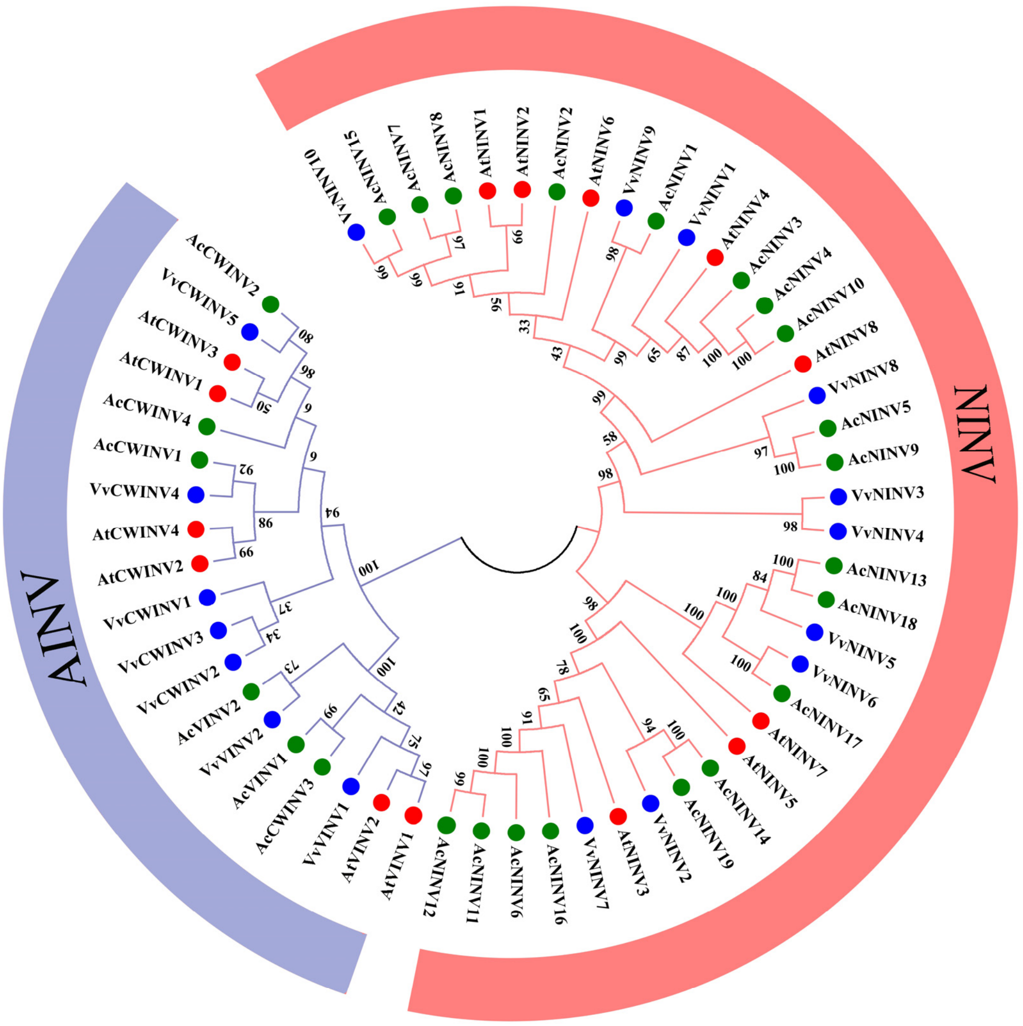

Figure S1. Phylogenetic relationships of members of the *INV* gene family in kiwifruit, *Arabidopsis thaliana*, and grape. Different colored graphs represent different plant species.

Table S3. Amino acid conserved sequence of kiwifruit *INV* family genes.

Motifs	Name
Motif1	WEELVGEMPLKICYPALESHEWRIITGCDPKNTPWSYHNGGSWPTLLWLL
Motif2	ERPEVQTGIRLILNLCLSEGFDTFPTLLVADGSCMIDRRMGIYGYPJEIQ
Motif3	KRLHALSFHIRSYYWLDMKQJNDIYRYKTEEYSHTAVNKFNVYPDSIPEW
Motif4	LNGEPEIVKNFLLKTLQLQSWEKTVDCFSLGZGLMPASFKVLHDPLRGTD
Motif5	CCIKTGRPZIARRAIELAEKRLSKDGWPEYYDGKLGRYIGKQARKYQTWS
Motif6	DFMPTRGGYLIGNVSPARMDFRWFTLGNCIAILSSLATPEQSEAIMDLIE
Motif7	ADFGESAIGRVAPVDSGFWWIILLRAYTK
Motif8	SPMEAEAWELLRRSVVYYCGQPVGTIACNDPADEQ
Motif9	IAGYLVAKMLLEBPSHLGMJSLEEDKZLK
Motif10	LNYDQVFVRDFVPSA
Motif11	ALFFMALRCALEMLKHDAEGK
Motif12	IASVASNVRNYSTSVETRVNEKNFERIYVPGGLNVKPLIVERIDKDENIG

Table S4. Distribution of ka/ks in the *Arabidopsis* and kiwifruit *INV* gene families.

Homologous gene pairs	Ka	Ks	Ka/Ks
AtNINV4 & *AcNINV1*	0.199086	4.4286	0.0449547
AtNINV4 & *AcNINV3*	0.171163	4.4382	0.0385659
AtNINV4 & *AcNINV4*	0.1672	4.4494	0.037578
AtNINV4 & *AcNINV10*	0.159632	4.98962	0.0319928
AtNINV5 & *AcNINV12*	0.249055	4.27392	0.0582731
AtNINV7 & *AcNINV13*	0.242477	2.49691	0.0971109
AtNINV7 & *AcNINV18*	0.262551	1.95435	0.134342
AtNINV8 & *AcNINV2*	0.14058	4.24592	0.0331095
AtVINV2 & *AcVINV1*	0.376439	2.75975	0.136403
AtVINV2 & *AcVINV2*	0.401874	4.63205	0.0867593
AtCWINV4 & *AcCWINV1*	0.334646	4.47336	0.0748087

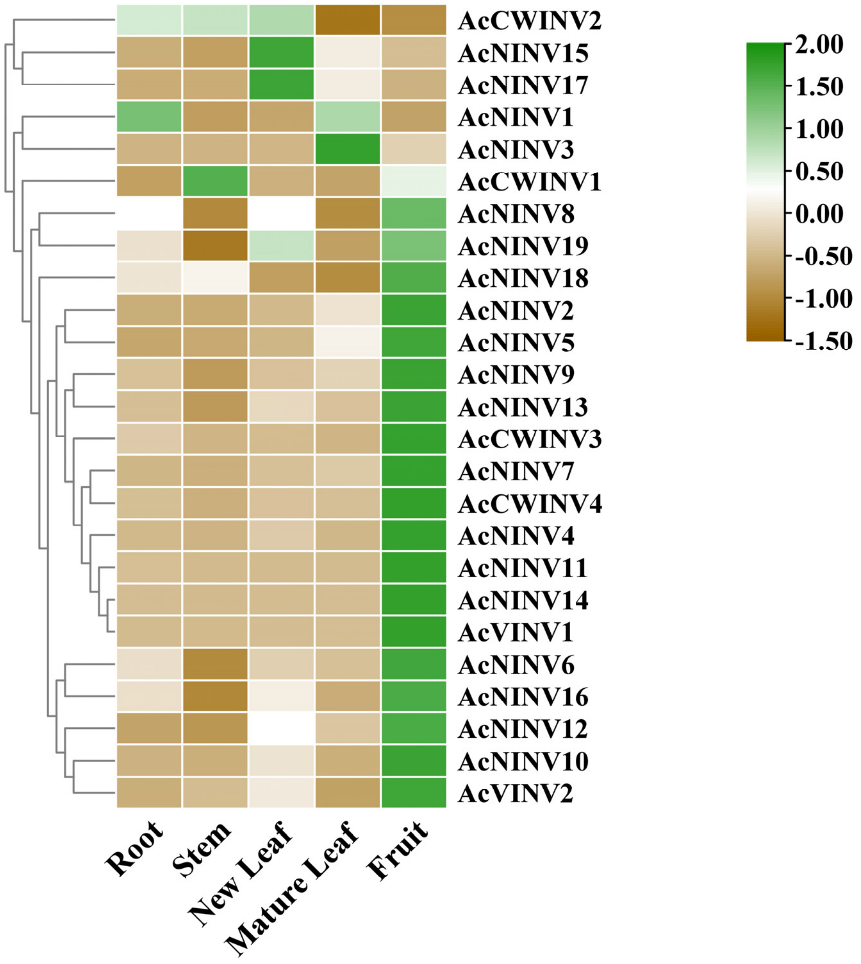

Figure S2. Expression of *AcINV* genes in different tissues of kiwifruit.

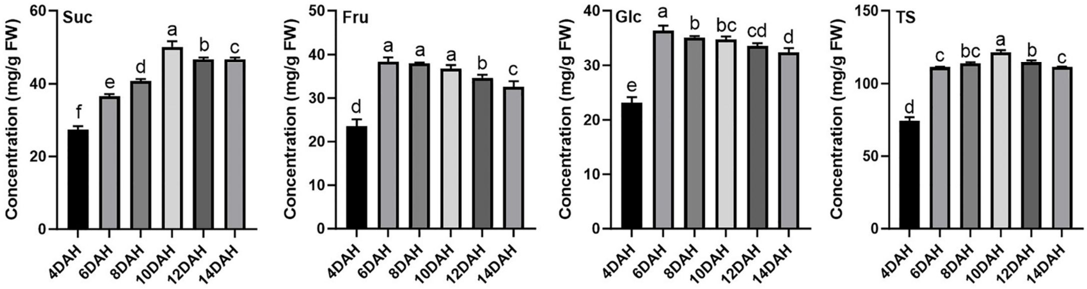

Figure S3. Sugar content of kiwifruit at different stages after harvest.

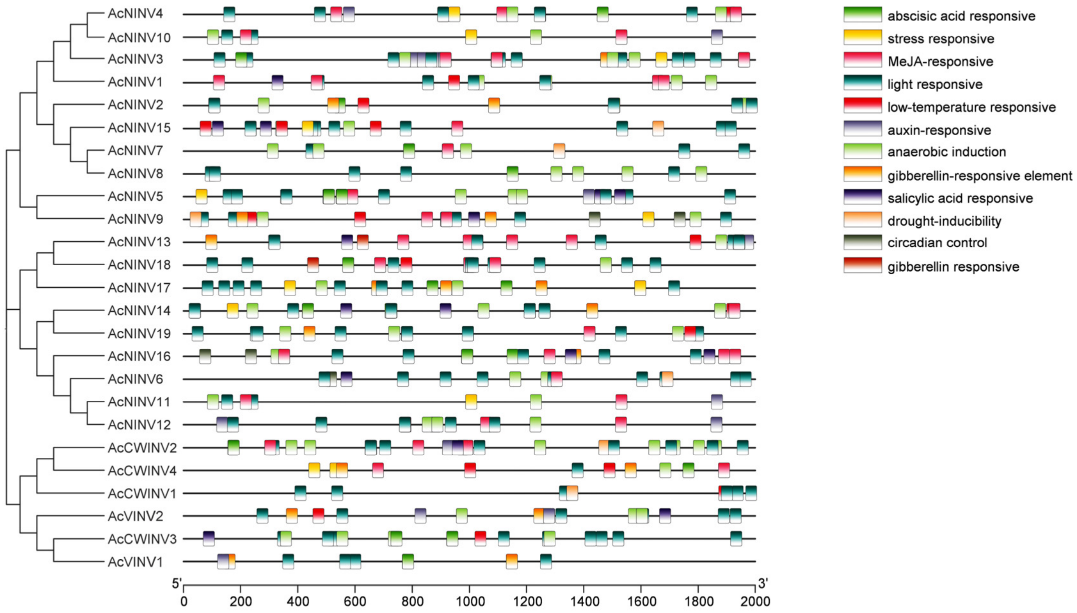

Figure S4. Cis-acting elements of kiwifruit *INV* gene family.
